# *cis*-Bis(2,2′-bipyridine-κ^2^ *N*,*N*′)dichlorido­iron(III) perchlorate

**DOI:** 10.1107/S1600536811016035

**Published:** 2011-05-07

**Authors:** Zhi-Fang Zhang

**Affiliations:** a School of Chemistry and Chemical Engineering, Yulin University, Yulin 719000, People’s Republic of China

## Abstract

In the crystal structure of the title compound, [FeCl_2_(C_10_H_8_N_2_)_2_]ClO_4_, the coordination around the Fe^III^ atom is approximately octa­hedral. The equatorial positions are occupied by two N atoms from two 2,2′-bipyridyl ligands [Fe—N = 2.121 (5) and 2.147 (5) Å] and two Cl atoms [Fe—Cl = 2.220 (2) and 2.2074 (18) Å]. Weak inter­molecular C—H⋯O and C—H⋯Cl hydrogen bonds and C—H⋯π inter­actions consolidate the crystal packing.

## Related literature

For the use of bipyridine and analogous ligands in the formation of transition metal complexes, see: Constable (1989 ▶). For applications of related compounds, see: Constable & Steel (1989 ▶); Steel *et al.* (1990 ▶). For related structures, see: Amani *et al.* (2007 ▶); Figgis *et al.* (1983 ▶).
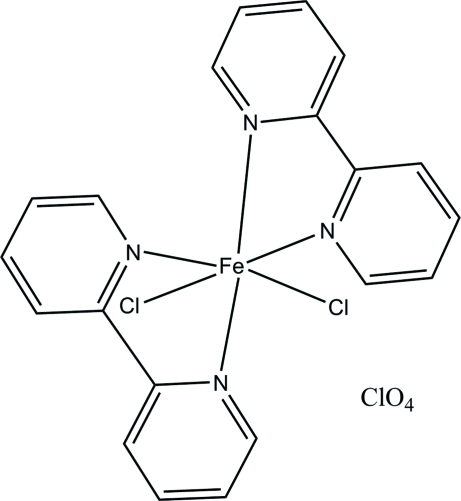


## Experimental

### 

#### Crystal data

[FeCl_2_(C_10_H_8_N_2_)_2_]ClO_4_
*M* *_r_* = 538.57
Orthorhombic, 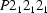

*a* = 10.891 (2) Å
*b* = 11.522 (2) Å
*c* = 16.990 (3) Å
*V* = 2132.1 (7) Å^3^
*Z* = 4
Mo *K*α radiation
μ = 1.12 mm^−1^
*T* = 295 K
0.34 × 0.29 × 0.24 mm

#### Data collection

Bruker APEXII CCD area-detector diffractometer
Absorption correction: multi-scan (*SADABS*; Sheldrick, 2003 ▶) *T*_min_ = 0.702, *T*_max_ = 0.775
5914 measured reflections
3534 independent reflections
2810 reflections with *I* > 2σ(*I*)
*R*_int_ = 0.038

#### Refinement

*R*[*F* ^2^ > 2σ(*F* ^2^)] = 0.063
*wR*(*F* ^2^) = 0.125
*S* = 1.08
3534 reflections
289 parameters
H-atom parameters constrained
Δρ_max_ = 0.75 e Å^−3^
Δρ_min_ = −0.42 e Å^−3^
Absolute structure: Flack (1983 ▶), 1419 Friedel pairs
Flack parameter: 0.05 (3)

### 

Data collection: *SMART* (Bruker, 2001 ▶); cell refinement: *SAINT-Plus* (Bruker, 2003 ▶); data reduction: *SAINT-Plus*; program(s) used to solve structure: *SHELXTL* (Sheldrick, 2008 ▶); program(s) used to refine structure: *SHELXTL*; molecular graphics: *SHELXTL*; software used to prepare material for publication: *SHELXTL*.

## Supplementary Material

Crystal structure: contains datablocks I, global. DOI: 10.1107/S1600536811016035/zq2098sup1.cif

Structure factors: contains datablocks I. DOI: 10.1107/S1600536811016035/zq2098Isup2.hkl

Additional supplementary materials:  crystallographic information; 3D view; checkCIF report

## Figures and Tables

**Table 1:** Hydrogen-bond geometry (Å, °) *Cg*4 is the centroid of the N2,C6–C10 ring.

*D*—H⋯*A*	*D*—H	H⋯*A*	*D*⋯*A*	*D*—H⋯*A*
C3—H3⋯O2	0.93	2.52	3.140 (11)	124
C7—H7⋯O3^i^	0.93	2.55	3.239 (9)	131
C8—H8⋯O2^ii^	0.93	2.30	3.152 (9)	152
C13—H13⋯O2^iii^	0.93	2.54	3.423 (10)	158
C18—H18⋯O4^iv^	0.93	2.51	3.387 (10)	158
C10—H10⋯Cl3	0.93	2.71	3.308 (7)	122
C20—H20⋯Cl2	0.93	2.79	3.382 (7)	123
C11—H11⋯*Cg*4	0.93	2.90	3.705 (8)	146

Symmetry codes: (i) 
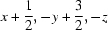
; (ii) 
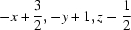
; (iii) 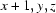; (iv) 
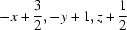
.

**Fig. 1.**
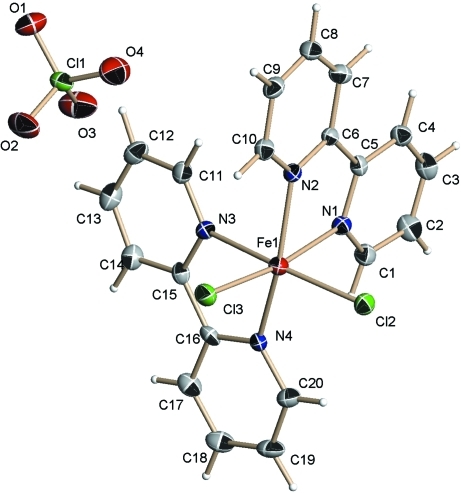
The molecular structure of the title compound showing thermal ellipsoids at the 30% probability level.

## supplementary crystallographic information

### Comment

Bipyridine and analogous ligands such as phenanthroline are commonly used in the formation of different complexes with a general variety of transition metals (Constable, 1989). Studies of these transition metal complexes are important in understanding electron transfer processes, mixed valence complexes, magnetic coupling and magnetic transitions (Constable *et al.*, 1989; Steel *et al.*, 1990). Although bipyridine coordination to iron has been widely investigated, most complexes are iron(II) complexes, little attention has been paid to bipyridine iron(III) complexes. In order to expand this field, the title compound has been synthesized, and its crystal structure is reported herein.

The molecular structure of the title compound (I) is shown in Fig. 1. The crystal is composed of *cis*-[Fe^III^(bipy)_2_C1_2_]^+^ cations and [C1O_4_]^-^ anions. The Fe^III^ atom is coordinated by two Cl anions and four N atoms from two 2,2'-bipyridyl ligands within a distorted octahedral geometry. The six-coordinate molecule is the *cis-cis* isomer considering the positions of the chlorine and pyridyl nitrogen atoms. The four Fe—N bond lengths [2.087 (4)–2.147 (5) Å] were similar and consistent with those reported earlier (Amani *et al.*, 2007; Figgis *et al.*, 1983). The distortion from a perfect octahedral geometry was primarily a consequence of the small bite-angle of the chelating ligands, which led to acute N1—Fe—N2 and N3—Fe—N4 angles of 75.96 (19)° and 75.4 (2)°, respectively.

Intermolecular C—H···O, C—H···Cl hydrogen bonds and C—H···π interactions stabilize the crystal structure (Table 1).

### Experimental

All reagents were obtained from commercial sources and used without further purification. 2,2'-Bipyridine (0.312 g, 2.0 mmol) and NaClO_4_ (0.122 g,1.0 mmol) were added to a solution of FeCl_3_.6H_2_O (0.270 g, 1.0 mmol) in methanol (30 ml), and the solution was stirred at 60–65 ^o^C for 3 h. A red-brown precipitate was obtained. After filtration, the red-brown filtrate was allowed to stand at room temperature for two weeks to give red-brown block-shaped crystals suitable for X-ray analysis. Elemental analysis for C_20_H_16_Cl_3_FeN_4_O_4_: C 44.60, H 2.99, N 10.40 %; found: C 44.52, H 3.03, N 10.39 %.

### Refinement

All C-bound H atoms were positioned geometrically and treated as riding, with C—H = 0.93Å and *U*_iso_(H) =1.2*U*_eq_(C).

### Crystal data

**Table:**

[FeCl_2_(C_10_H_8_N_2_)_2_]ClO_4_	*F*(000) = 1092
*M**_r_* = 538.57	*D*_x_ = 1.678 Mg m^−^^3^
Orthorhombic, *P*2_1_2_1_2_1_	Mo *K*α radiation, λ = 0.71073 Å
Hall symbol: P 2ac 2ab	Cell parameters from 1783 reflections
*a* = 10.891 (2) Å	θ = 2.4–25.9°
*b* = 11.522 (2) Å	µ = 1.12 mm^−^^1^
*c* = 16.990 (3) Å	*T* = 295 K
*V* = 2132.1 (7) Å^3^	Block, red-brown
*Z* = 4	0.34 × 0.29 × 0.24 mm

### Data collection

**Table:**

Bruker APEXII CCD area-detector diffractometer	3534 independent reflections
Radiation source: fine-focus sealed tube	2810 reflections with *I* > 2σ(*I*)
graphite	*R*_int_ = 0.038
φ and ω scans	θ_max_ = 25.0°, θ_min_ = 3.0°
Absorption correction: multi-scan (*SADABS*; Sheldrick, 2003)	*h* = −7→12
*T*_min_ = 0.702, *T*_max_ = 0.775	*k* = −12→13
5914 measured reflections	*l* = −20→17

### Refinement

**Table:**

Refinement on *F*^2^	Secondary atom site location: difference Fourier map
Least-squares matrix: full	Hydrogen site location: inferred from neighbouring sites
*R*[*F*^2^ > 2σ(*F*^2^)] = 0.063	H-atom parameters constrained
*wR*(*F*^2^) = 0.125	*w* = 1/[σ^2^(*F*_o_^2^) + (0.043*P*)^2^ + 0.4377*P*] where *P* = (*F*_o_^2^ + 2*F*_c_^2^)/3
*S* = 1.08	(Δ/σ)_max_ = 0.001
3534 reflections	Δρ_max_ = 0.75 e Å^−^^3^
289 parameters	Δρ_min_ = −0.42 e Å^−^^3^
0 restraints	Absolute structure: Flack (1983), 1419 Friedel pairs
Primary atom site location: structure-invariant direct methods	Flack parameter: 0.05 (3)

### Special details

**Table:**

Geometry. All e.s.d.'s (except the e.s.d. in the dihedral angle between two l.s. planes) are estimated using the full covariance matrix. The cell e.s.d.'s are taken into account individually in the estimation of e.s.d.'s in distances, angles and torsion angles; correlations between e.s.d.'s in cell parameters are only used when they are defined by crystal symmetry. An approximate (isotropic) treatment of cell e.s.d.'s is used for estimating e.s.d.'s involving l.s. planes.
Refinement. Refinement of *F*^2^ against ALL reflections. The weighted *R*-factor *wR* and goodness of fit *S* are based on *F*^2^, conventional *R*-factors *R* are based on *F*, with *F* set to zero for negative *F*^2^. The threshold expression of *F*^2^ > σ(*F*^2^) is used only for calculating *R*-factors(gt) *etc*. and is not relevant to the choice of reflections for refinement. *R*-factors based on *F*^2^ are statistically about twice as large as those based on *F*, and *R*- factors based on ALL data will be even larger.

### Fractional atomic coordinates and isotropic or equivalent isotropic displacement parameters (Å^2^)

**Table:**

	*x*	*y*	*z*	*U*_iso_*/*U*_eq_	
Fe1	0.97563 (9)	0.23098 (7)	0.13357 (5)	0.0557 (3)	
Cl1	0.4647 (2)	0.80918 (14)	0.13791 (10)	0.0674 (5)	
Cl2	0.79726 (18)	0.13772 (13)	0.13412 (11)	0.0693 (5)	
Cl3	1.10061 (18)	0.08017 (14)	0.13284 (11)	0.0722 (5)	
O1	0.5403 (7)	0.8608 (5)	0.0835 (3)	0.111 (2)	
O2	0.5221 (7)	0.7174 (5)	0.1726 (3)	0.127 (2)	
O3	0.4321 (8)	0.8861 (5)	0.1958 (3)	0.139 (3)	
O4	0.3639 (7)	0.7708 (8)	0.0978 (4)	0.164 (3)	
N1	0.8727 (5)	0.3863 (4)	0.1221 (3)	0.0511 (13)	
N2	0.9729 (5)	0.2621 (4)	0.0126 (3)	0.0499 (12)	
N3	1.1301 (5)	0.3461 (4)	0.1420 (3)	0.0551 (13)	
N4	0.9974 (5)	0.2596 (4)	0.2546 (3)	0.0529 (13)	
C1	0.8236 (7)	0.4446 (6)	0.1798 (4)	0.067 (2)	
H1	0.8394	0.4209	0.2311	0.080*	
C2	0.7503 (8)	0.5385 (6)	0.1681 (5)	0.079 (3)	
H2	0.7126	0.5762	0.2101	0.095*	
C3	0.7341 (8)	0.5753 (6)	0.0926 (5)	0.076 (2)	
H3	0.6866	0.6406	0.0823	0.092*	
C4	0.7870 (8)	0.5169 (6)	0.0333 (4)	0.065 (2)	
H4	0.7762	0.5419	−0.0183	0.078*	
C5	0.8558 (6)	0.4220 (5)	0.0484 (4)	0.0481 (15)	
C6	0.9143 (6)	0.3543 (5)	−0.0115 (3)	0.0475 (15)	
C7	0.9110 (8)	0.3798 (6)	−0.0911 (4)	0.067 (2)	
H7	0.8674	0.4442	−0.1086	0.081*	
C8	0.9698 (7)	0.3129 (7)	−0.1431 (4)	0.073 (2)	
H8	0.9702	0.3321	−0.1963	0.087*	
C9	1.0269 (7)	0.2198 (6)	−0.1181 (4)	0.0691 (19)	
H9	1.0661	0.1710	−0.1538	0.083*	
C10	1.0287 (7)	0.1949 (5)	−0.0396 (4)	0.0604 (17)	
H10	1.0701	0.1291	−0.0223	0.072*	
C11	1.1910 (7)	0.3941 (6)	0.0843 (4)	0.0658 (19)	
H11	1.1659	0.3775	0.0333	0.079*	
C12	1.2875 (9)	0.4658 (6)	0.0934 (5)	0.079 (2)	
H12	1.3255	0.5006	0.0504	0.094*	
C13	1.3264 (8)	0.4850 (6)	0.1671 (5)	0.079 (2)	
H13	1.3935	0.5331	0.1762	0.095*	
C14	1.2681 (8)	0.4344 (6)	0.2280 (4)	0.070 (2)	
H14	1.2965	0.4458	0.2790	0.084*	
C15	1.1680 (7)	0.3669 (5)	0.2151 (4)	0.0533 (16)	
C16	1.0945 (7)	0.3178 (5)	0.2775 (4)	0.0564 (18)	
C17	1.1226 (8)	0.3310 (5)	0.3553 (4)	0.072 (2)	
H17	1.1922	0.3722	0.3703	0.087*	
C18	1.0481 (10)	0.2833 (7)	0.4102 (4)	0.085 (3)	
H18	1.0670	0.2904	0.4633	0.102*	
C19	0.9466 (9)	0.2257 (7)	0.3879 (4)	0.086 (3)	
H19	0.8932	0.1947	0.4251	0.104*	
C20	0.9236 (8)	0.2136 (6)	0.3085 (4)	0.078 (2)	
H20	0.8548	0.1722	0.2923	0.094*	

### Atomic displacement parameters (Å^2^)

**Table:**

	*U*^11^	*U*^22^	*U*^33^	*U*^12^	*U*^13^	*U*^23^
Fe1	0.0563 (6)	0.0609 (5)	0.0499 (5)	0.0006 (5)	−0.0004 (5)	0.0042 (4)
Cl1	0.0817 (14)	0.0688 (9)	0.0518 (9)	0.0106 (10)	−0.0023 (11)	0.0058 (9)
Cl2	0.0587 (11)	0.0678 (9)	0.0814 (11)	−0.0092 (8)	−0.0010 (11)	0.0118 (10)
Cl3	0.0703 (13)	0.0697 (9)	0.0765 (11)	0.0135 (9)	−0.0031 (11)	0.0088 (10)
O1	0.126 (6)	0.117 (4)	0.089 (3)	−0.022 (4)	0.033 (4)	0.012 (3)
O2	0.152 (7)	0.112 (4)	0.117 (4)	0.065 (5)	0.009 (4)	0.036 (3)
O3	0.215 (9)	0.108 (4)	0.093 (4)	0.054 (5)	0.044 (5)	−0.004 (3)
O4	0.128 (7)	0.259 (9)	0.104 (4)	−0.074 (7)	−0.041 (5)	0.035 (5)
N1	0.054 (4)	0.054 (3)	0.045 (3)	−0.005 (2)	0.001 (3)	−0.006 (3)
N2	0.047 (3)	0.056 (3)	0.047 (3)	0.004 (3)	0.005 (3)	−0.001 (2)
N3	0.051 (3)	0.056 (3)	0.058 (3)	−0.007 (3)	0.003 (3)	0.009 (3)
N4	0.043 (3)	0.067 (3)	0.049 (3)	0.006 (3)	−0.001 (2)	0.009 (2)
C1	0.065 (6)	0.077 (4)	0.058 (4)	0.002 (4)	−0.002 (4)	−0.004 (4)
C2	0.092 (7)	0.064 (4)	0.081 (5)	0.014 (5)	−0.005 (5)	−0.025 (4)
C3	0.076 (7)	0.055 (4)	0.099 (6)	0.010 (4)	−0.010 (5)	−0.011 (5)
C4	0.067 (6)	0.057 (4)	0.071 (4)	−0.002 (4)	−0.004 (4)	0.008 (4)
C5	0.044 (4)	0.038 (3)	0.062 (4)	−0.003 (3)	−0.007 (3)	−0.002 (3)
C6	0.043 (4)	0.050 (3)	0.049 (3)	−0.005 (3)	−0.003 (3)	0.005 (3)
C7	0.077 (6)	0.068 (4)	0.058 (4)	−0.015 (4)	−0.008 (4)	0.010 (4)
C8	0.070 (5)	0.101 (5)	0.047 (4)	−0.003 (5)	−0.001 (4)	0.006 (4)
C9	0.061 (5)	0.091 (5)	0.055 (4)	−0.013 (5)	0.008 (4)	−0.012 (4)
C10	0.056 (5)	0.064 (4)	0.060 (4)	0.001 (4)	0.001 (4)	−0.008 (3)
C11	0.054 (5)	0.068 (4)	0.075 (5)	0.000 (4)	0.002 (4)	0.001 (4)
C12	0.080 (7)	0.062 (4)	0.094 (6)	−0.015 (4)	0.017 (5)	0.007 (5)
C13	0.068 (6)	0.067 (4)	0.102 (6)	−0.008 (4)	0.014 (5)	−0.023 (5)
C14	0.068 (6)	0.067 (4)	0.075 (5)	−0.001 (4)	−0.002 (4)	−0.019 (4)
C15	0.048 (5)	0.051 (3)	0.062 (4)	0.002 (3)	−0.009 (3)	−0.009 (3)
C16	0.066 (5)	0.051 (4)	0.051 (4)	0.016 (4)	−0.017 (4)	0.003 (3)
C17	0.088 (6)	0.066 (4)	0.063 (4)	0.004 (4)	−0.018 (5)	−0.012 (4)
C18	0.122 (9)	0.084 (5)	0.049 (4)	0.002 (6)	−0.008 (5)	−0.005 (4)
C19	0.115 (8)	0.096 (6)	0.048 (4)	0.006 (6)	0.011 (4)	0.017 (4)
C20	0.079 (6)	0.097 (5)	0.060 (4)	−0.008 (5)	0.007 (4)	0.014 (4)

### Geometric parameters (Å, °)

**Table:**

Fe1—N2	2.087 (4)	C5—C6	1.432 (8)
Fe1—N4	2.096 (5)	C6—C7	1.385 (8)
Fe1—N1	2.121 (5)	C7—C8	1.336 (9)
Fe1—N3	2.147 (5)	C7—H7	0.9300
Fe1—Cl3	2.2074 (18)	C8—C9	1.310 (9)
Fe1—Cl2	2.220 (2)	C8—H8	0.9300
Cl1—O2	1.363 (5)	C9—C10	1.366 (8)
Cl1—O4	1.366 (7)	C9—H9	0.9300
Cl1—O3	1.371 (5)	C10—H10	0.9300
Cl1—O1	1.373 (6)	C11—C12	1.346 (11)
N1—C1	1.302 (8)	C11—H11	0.9300
N1—C5	1.332 (7)	C12—C13	1.340 (11)
N2—C6	1.306 (7)	C12—H12	0.9300
N2—C10	1.324 (7)	C13—C14	1.346 (9)
N3—C11	1.306 (8)	C13—H13	0.9300
N3—C15	1.330 (7)	C14—C15	1.358 (9)
N4—C16	1.311 (8)	C14—H14	0.9300
N4—C20	1.329 (8)	C15—C16	1.444 (9)
C1—C2	1.360 (10)	C16—C17	1.365 (8)
C1—H1	0.9300	C17—C18	1.353 (10)
C2—C3	1.362 (10)	C17—H17	0.9300
C2—H2	0.9300	C18—C19	1.344 (11)
C3—C4	1.342 (9)	C18—H18	0.9300
C3—H3	0.9300	C19—C20	1.378 (9)
C4—C5	1.350 (9)	C19—H19	0.9300
C4—H4	0.9300	C20—H20	0.9300
			
N2—Fe1—N4	160.23 (18)	C4—C5—C6	123.6 (6)
N2—Fe1—N1	75.96 (19)	N2—C6—C7	119.5 (6)
N4—Fe1—N1	90.98 (18)	N2—C6—C5	116.0 (5)
N2—Fe1—N3	88.34 (19)	C7—C6—C5	124.5 (6)
N4—Fe1—N3	75.4 (2)	C8—C7—C6	120.7 (7)
N1—Fe1—N3	84.2 (2)	C8—C7—H7	119.6
N2—Fe1—Cl3	97.95 (16)	C6—C7—H7	119.6
N4—Fe1—Cl3	93.40 (14)	C9—C8—C7	119.1 (7)
N1—Fe1—Cl3	171.80 (16)	C9—C8—H8	120.5
N3—Fe1—Cl3	90.20 (15)	C7—C8—H8	120.5
N2—Fe1—Cl2	94.29 (15)	C8—C9—C10	119.7 (7)
N4—Fe1—Cl2	99.84 (16)	C8—C9—H9	120.1
N1—Fe1—Cl2	86.93 (15)	C10—C9—H9	120.1
N3—Fe1—Cl2	169.85 (15)	N2—C10—C9	121.6 (6)
Cl3—Fe1—Cl2	99.13 (8)	N2—C10—H10	119.2
O2—Cl1—O4	109.4 (5)	C9—C10—H10	119.2
O2—Cl1—O3	108.0 (4)	N3—C11—C12	124.8 (7)
O4—Cl1—O3	111.0 (6)	N3—C11—H11	117.6
O2—Cl1—O1	110.7 (4)	C12—C11—H11	117.6
O4—Cl1—O1	106.7 (4)	C13—C12—C11	117.1 (8)
O3—Cl1—O1	111.0 (4)	C13—C12—H12	121.4
C1—N1—C5	119.5 (6)	C11—C12—H12	121.4
C1—N1—Fe1	125.7 (4)	C12—C13—C14	119.8 (8)
C5—N1—Fe1	114.8 (4)	C12—C13—H13	120.1
C6—N2—C10	119.3 (5)	C14—C13—H13	120.1
C6—N2—Fe1	117.1 (4)	C13—C14—C15	120.2 (7)
C10—N2—Fe1	123.5 (4)	C13—C14—H14	119.9
C11—N3—C15	117.8 (6)	C15—C14—H14	119.9
C11—N3—Fe1	127.5 (5)	N3—C15—C14	120.2 (7)
C15—N3—Fe1	114.6 (4)	N3—C15—C16	116.3 (6)
C16—N4—C20	119.2 (6)	C14—C15—C16	123.5 (6)
C16—N4—Fe1	117.6 (4)	N4—C16—C17	121.7 (7)
C20—N4—Fe1	123.0 (5)	N4—C16—C15	115.4 (5)
N1—C1—C2	122.8 (7)	C17—C16—C15	122.9 (7)
N1—C1—H1	118.6	C18—C17—C16	119.2 (7)
C2—C1—H1	118.6	C18—C17—H17	120.4
C1—C2—C3	117.5 (7)	C16—C17—H17	120.4
C1—C2—H2	121.3	C19—C18—C17	120.0 (7)
C3—C2—H2	121.3	C19—C18—H18	120.0
C4—C3—C2	119.7 (7)	C17—C18—H18	120.0
C4—C3—H3	120.1	C18—C19—C20	118.4 (8)
C2—C3—H3	120.1	C18—C19—H19	120.8
C3—C4—C5	120.1 (7)	C20—C19—H19	120.8
C3—C4—H4	119.9	N4—C20—C19	121.6 (8)
C5—C4—H4	119.9	N4—C20—H20	119.2
N1—C5—C4	120.3 (6)	C19—C20—H20	119.2
N1—C5—C6	116.1 (5)		

### Hydrogen-bond geometry (Å, °)

**Table:**

Cg4 is the centroid of the N2,C6–C10 ring.

**Table:**

*D*—H···*A*	*D*—H	H···*A*	*D*···*A*	*D*—H···*A*
C3—H3···O2	0.93	2.52	3.140 (11)	124
C7—H7···O3^i^	0.93	2.55	3.239 (9)	131
C8—H8···O2^ii^	0.93	2.30	3.152 (9)	152
C13—H13···O2^iii^	0.93	2.54	3.423 (10)	158
C18—H18···O4^iv^	0.93	2.51	3.387 (10)	158
C10—H10···Cl3	0.93	2.71	3.308 (7)	122
C20—H20···Cl2	0.93	2.79	3.382 (7)	123
C11—H11···Cg4	0.93	2.90	3.705 (8)	146

Symmetry codes:  (i) *x*+1/2, −*y*+3/2, −*z*; (ii) −*x*+3/2, −*y*+1, *z*−1/2; (iii) *x*+1, *y*, *z*; (iv) −*x*+3/2, −*y*+1, *z*+1/2.
